# Rising Incidence of Neuroendocrine Neoplasms in Northern Switzerland—Data From the Cancer Registry

**DOI:** 10.1210/jendso/bvaf194

**Published:** 2025-11-27

**Authors:** Alessa Fischer, Miriam Wanner, Flurina Suter, Dimitri Korol, Constanze Hantel, Felix Beuschlein, Sena Blümel, Ralph Fritsch, Andreas Wicki, Sabine Rohrmann, Svenja Nölting

**Affiliations:** Department of Endocrinology, Diabetology and Clinical Nutrition, University Hospital Zurich and University of Zurich, Zurich CH-8091, Switzerland; Cancer Registry of the Canton Zurich, Zug, Schaffhausen and Schwyz, University Hospital Zurich, Zurich CH-8091, Switzerland; Cancer Registry of the Canton Zurich, Zug, Schaffhausen and Schwyz, University Hospital Zurich, Zurich CH-8091, Switzerland; Division of Chronic Disease Epidemiology, Epidemiology, Biostatistics, and Prevention Institute, University of Zurich, Zurich CH-8091, Switzerland; Cancer Registry of the Canton Zurich, Zug, Schaffhausen and Schwyz, University Hospital Zurich, Zurich CH-8091, Switzerland; Division of Chronic Disease Epidemiology, Epidemiology, Biostatistics, and Prevention Institute, University of Zurich, Zurich CH-8091, Switzerland; Department of Endocrinology, Diabetology and Clinical Nutrition, University Hospital Zurich and University of Zurich, Zurich CH-8091, Switzerland; Department of Endocrinology, Diabetology and Clinical Nutrition, University Hospital Zurich and University of Zurich, Zurich CH-8091, Switzerland; Department of Medicine IV, University Hospital, Ludwig-Maximilians-University Munich, Munich 80336, Germany; The LOOP Zurich–Medical Research Center, Zurich 8044, Switzerland; Department of Gastroenterology and Hepatology, University Hospital Zurich, Zurich CH-8091, Switzerland; Department of Medical Oncology and Hematology, University Hospital Zurich, Zurich CH-8091, Switzerland; Cancer Registry of the Canton Zurich, Zug, Schaffhausen and Schwyz, University Hospital Zurich, Zurich CH-8091, Switzerland; Department of Medical Oncology and Hematology, University Hospital Zurich, Zurich CH-8091, Switzerland; Cancer Registry of the Canton Zurich, Zug, Schaffhausen and Schwyz, University Hospital Zurich, Zurich CH-8091, Switzerland; Division of Chronic Disease Epidemiology, Epidemiology, Biostatistics, and Prevention Institute, University of Zurich, Zurich CH-8091, Switzerland; Department of Endocrinology, Diabetology and Clinical Nutrition, University Hospital Zurich and University of Zurich, Zurich CH-8091, Switzerland; Department of Medicine IV, University Hospital, Ludwig-Maximilians-University Munich, Munich 80336, Germany

**Keywords:** gastroenteropancreatic, pulmonary, NEN, PPGL, incidence, adrenocortical carcinoma

## Abstract

**Context:**

Neuroendocrine neoplasms (NENs) are a heterogeneous group of tumors that arise in multiple organs and encompass pheochromocytomas/paragangliomas (PPGLs). Adrenocortical carcinoma (ACC), though distinct, is a rare endocrine malignancy with a poor prognosis. We analyzed incidence and survival trends across NENs and ACC over 4 decades.

**Methods:**

We conducted a population-based study using cancer registry data from the Canton of Zurich (1980-2022). NENs were classified by site and histology. Age-standardized incidence rates (ASIRs) were calculated per 100,000 person-years (European standard population). Joinpoint regression estimated the annual percent change (APC) for each trend segment and the average APC (AAPC) for the period.

**Results:**

A total of 2723 patients with a diagnosis of a NEN (n = 2647) or ACC (n = 76) between 1980 and 2022 were extracted from the database. ASIR of gastrointestinal NENs rose from 1.4 in 1980 to 11.3 per 100,000 in 2022 [AAPC +5.07%, 95% confidence interval (CI) 4.20-6.50%], with the most pronounced increases in rectal and appendiceal NENs, particularly since the early 2000s. Pancreatic NEN incidence also increased, especially from 2004 to 2022 (0.6-2.7 per 100,000; APC 5.36%, 95% CI 3.13-16.97%). ASIR of PPGLs rose from 0 in 1980 to 0.4 per 100,000 by 2022, while the ASIRs of ACC remained stable with ASIR of 0.2 per 100,000 in 2022.

**Conclusion:**

The incidence of gastroenteropancreatic NENs and PPGLs continues to rise, with a pronounced acceleration since the early 2000s. These trends underscore the need for a deeper understanding of risk factors underlying NEN development.

Neuroendocrine neoplasms (NENs) represent a diverse group of tumors originating from endocrine tissues located throughout the body. NENs can be found in the gastrointestinal system, the pancreas, the lung, the thymus, or the thyroid [medullary thyroid carcinomas (MTCs)]. Based on histology (morphology and proliferation), NENs are categorized in well-differentiated neuroendocrine tumors (NETs) G1, G2, or G3 depending on their proliferation (mitotic index, Ki-67 index) and poorly differentiated neuroendocrine carcinomas (NECs) [1]. NECs represent approximately 10% of all NENs depending on the organ they arise from [1].

Tumors arising from the sympathetic and parasympathetic paraganglia (paragangliomas) or from the adrenal medulla (pheochromocytomas; intra-adrenal paragangliomas) collectively known as pheochromocytomas/paragangliomas (PPGLs), also belong to the family of NENs [2, 3]. Adrenocortical carcinomas (ACCs), though not NENs, are very rare endocrine malignancies that arise from the cortex of the adrenal gland and generally have a poor prognosis [4].

Surgery is the only curative treatment option for most NENs, ACCs, and PPGLs [4, 5]. However, at the time of diagnosis, approximately 40% to 45% of gastroenteropancreatic (GEP) NENs have already metastasized to other organs, necessitating systemic treatment approaches [5].

Globally, an increase in NEN incidence has been reported since the early 2000s [6-11]. In Europe, Swiss cancer registry data from Vaud and Neuchâtel (1976–2016) reported an average annual rise in GEP-NEN incidence by 1.7% in men and 1.3% in women [12]. Data from Germany showed a doubling of GEP-NEN incidence from 2.2 to 4.8 per 100,000 between 2005 and 2019 [7]. In the United States, GEP- and pulmonary NEN incidence increased more than 5-fold between the 1970s and 2021, reaching up to 8.52 per 100,000 (2021), with prominent increases observed across almost all sites and stages and a shift toward earlier-stage diagnoses [6, 13]. In the present study, we analyzed incidence trends and survival across a comprehensive range of neuroendocrine neoplasms, including not only GEP-NENs but also pulmonary NENs, medullary thyroid carcinoma, and PPGLs and, in addition, reported separately on the non-NEN entity ACC using data from the cancer registry of the Canton of Zurich, the most populous region in Switzerland.

## Methods

This study retrospectively analyzed data from the population-based cancer registry of the Canton of Zurich. The Canton of Zurich represents approximately 18% of the total population of Switzerland (end of 2022: Zurich 1.578 million, Switzerland 8.815 million inhabitants) [14]. Within the cancer registry, data of every patient diagnosed with cancer whose registered residence was in the Canton of Zurich at the time of diagnosis have been collected since 1980 [15]. Patient and tumor information is derived from pathology laboratories, treating physicians, and hospitals. Cancer cases in the Canton of Zurich have been registered under a presumed consent framework, based on a 1980 decision by the Zurich Government Council and a general approval granted in 1995 by the Federal Commission of Experts for Professional Secrecy in Medical Research. All data used for this study were anonymized.

For this study, we analyzed patients diagnosed with NENs based on histology and registered in the cancer registry of Zurich between 1980 and 2022. Primary tumor site topography and morphology according to the International Classification of Diseases for Oncology (ICD-O), age at diagnosis, sex, and survival data were extracted from the registry in April 2025. The ICD-O topography code defines the tumor's anatomical site of origin, while the morphology code specifies its histological type and behavior.

NENs were categorized based on topography and morphology codes as follows (Table S1 [16]): esophageal NEN (C15), gastric NEN (C16), small intestinal NEN (C17), colonic NEN (C18, excluding C18.1; C19; C26), rectal NEN (C20), appendiceal NEN (C18.1), pancreatic NEN (C25), pulmonary carcinoid (C34), PPGL (C74.1; morphology code 8700 and C75.5; morphology code 8680), ACC (C74.0; morphology code 8370), MTC (C73.9; morphology codes 8345-8347), thymic carcinoid (C37.9; morphology codes 8240 and 8249), and mixed neuroendocrine-nonneuroendocrine neoplasms (MiNENs) of any site (morphology codes 8244 and 8245). Based on morphology independent of site, NETs were further differentiated into NETs G1 (morphology codes 8240) and G2/3 (8249; the distinction between grades 2 and 3 was not feasible due to the absence of proliferative indices); poorly differentiated NECs including small-cell NECs (code 8041), large-cell NECs (codes 8013 and 8243), and NECs not otherwise specified (code 8246); and MiNENs including codes 8154 (of the pancreas) and 8244/45. The group “other pancreatic NETs” comprised functioning pancreatic NETs such as insulinoma (code 8151), glucagonoma (code 8152), gastrinoma (code 8153), vipoma (code 8155), and nonfunctioning pancreatic endocrine tumor, well-differentiated, with grade not further specified (code 8150).

### Statistics

Annual incidence rates were calculated per 100,000 person-years and age-standardized to the European standard population using the direct method [17]. Population data from the Canton of Zurich (1981-2022) were used for standardization [14]. For 1980, population data of the Canton of Zurich were not available; therefore, the population from 1981 was used. Rates were stratified by tumor site and sex. Temporal trends were assessed using Joinpoint regression software [18], applying a grid search algorithm with constraints on minimum segment length and number of joinpoints [18, 19]. Heteroscedasticity was adjusted for by using standard errors. Average annual percent change (AAPC) with 95% confidence intervals (CIs) was estimated as a summary measure for the entire observation period (1980-2022). Annual percentage changes (APC) were estimated for each trend segment between two joinpoints.

The Kaplan–Meier method was used to estimate median overall survival (OS) stratified by tumor site with follow-up time defined as the time between diagnosis and death, loss of follow-up, or last follow-up, whichever occurred first. A log-rank test was performed to compare the survival distribution of subgroups. Statistical significance was determined at a 2-sided α of .05. Statistical analyses were conducted using the open-source statistics software R (version 4.3.2, R Foundation for Statistical Computing, Vienna, Austria) [20].

## Results

A total of 2723 patients with a diagnosis of a NEN or ACC between 1980 and 2022 were extracted from the database of the Cancer Registry of the Canton of Zurich. Thereof, 1363 (50.1%) were female. Gastrointestinal NENs were present in 1581 patients, with small-intestinal NENs being the most frequent (n = 626) within this group. Pancreatic NENs were present in 397 patients; 486 patients had a pulmonary carcinoid, 51 patients a PPGL, 76 patients an ACC, 64 patients an MTC, and 5 patients a thymic carcinoid. Sixty-three patients had mixed carcinomas at different sites and were therefore not included in further site-specific calculations. Characteristics of the study cohort are presented in Table 1.

**Table 1.** Characteristics of the study cohort

	Esophageal NEN	Gastric NEN	Small intestinal NEN	Colonic NEN	Rectal NEN	Appendiceal NEN	Pancreatic NEN	Pulmonary Carcinoid	PPGL	ACC	MTC	Thymic Carcinoid	Mixed Carcinoid	Total
n, patients (%)	15 (0.6)	144 (5.3)	626 (23)	147 (5.4)	235 (8.6)	414 (15.2)	397 (14.6)	486 (17.8)	51 (1.9)	76 (2.8)	64 (2.4)	5 (0.2)	63 (2.3)	2723 (100)
n, female	7	65	261	68	109	250	164	284	24	51	40	1	39	1363
n, male	8	79	365	79	126	164	233	202	27	25	24	4	24	1360
Age, median (min–max)	76 (58-87)	67 (25-90)	68 (25-96)	65 (21-97)	54 (18-90)	51.5 (9-94)	63 (13-90)	65.5 (20-99)	54 (20-81)	57.5 (1-83)	55 (6-82)	53 (26-82)	64 (37-87)	
No. of patients per age groups (years)														
<20	—	—	—	—	1	33	4	—	—	2	2	—	—	42
20-34	—	2	10	7	21	63	15	32	6	9	9	1	—	175
35-49	—	24	44	19	46	102	63	53	16	12	10	1	9	399
50-64	2	38	186	47	107	109	137	144	15	23	28	1	23	860
65-74	5	39	197	38	38	59	86	128	8	18	8	1	16	641
75-84	6	32	146	29	16	37	76	103	6	12	7	1	14	485
>84	2	9	43	7	6	11	16	26	—	—	—	—	1	121

Abbreviations: ACC, adrenocortical carcinoma; MTC, medullary thyroid carcinoma; NEN, neuroendocrine neoplasm; PPGL, pheochromocytoma/paraganglioma.

### Trends in Incidence of GEP-NENs

**Figure 1.**
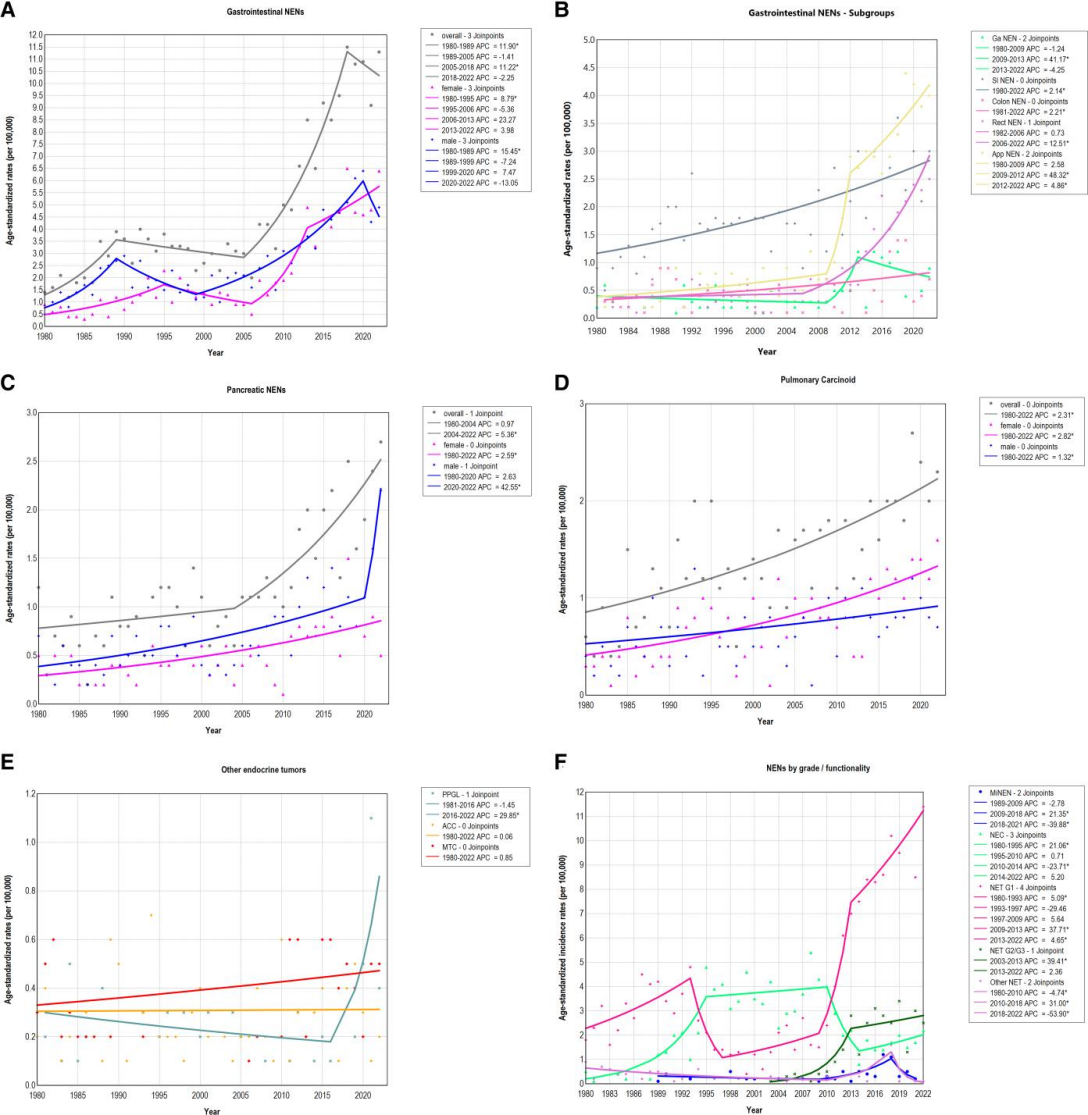
Trends in age-standardized incidence rates per 100,000 person-years of NENs by site and sex in the Canton of Zurich, 1980-2022. (A) Gastrointestinal NENs; (B) subgroups of gastrointestinal NENs (for esophageal NENs, no graphical analysis was possible due to the low number of patients); (C) pancreatic NENs; (D) pulmonary carcinoids; (E) other endocrine tumors; (F) by grade and functionality. Abbreviations: MiNEN, mixed neuroendocrine–nonneuroendocrine neoplasms; NEC, neuroendocrine carcinoma; NEN, neuroendocrine neoplasm; Other NET, functioning pancreatic neuroendocrine tumors and nonfunctioning pancreatic endocrine tumor with grade not further specified.

Overall, a significant increase in age-standardized incidence rates (ASIR) of gastrointestinal NENs was observed during the study period from 1.4 in 1980 to 11.3 per 100,000 person-years in 2022, corresponding to a significant AAPC of +5.07% (95% CI 4.20-6.50%). The APC increased most dramatically after 2005 from 3 to 11.5 per 100,000 in 2018 (APC 11.22%, 95% CI 8.99-21.80%), followed by a decline until 2022. The significant increase in ASIR of gastrointestinal NENs was observed for both sexes (Fig. 1A, Table 2).

**Table 2.** Summary of ASIR and AAPC for subgroups of NENs and ACC by site and sex (1980-2022)

Tumor type	Group	Number of cases	ASIR 1980 (per 100,000)	ASIR 2022 (per 100,000)	AAPC (%)	95% CI (%)
GI NEN	Overall	1581	1.4	11.3	5.07*^a^*	4.20-6.50
	Female	760	0.9	6.4	6.07*^a^*	4.96-8.01
	Male	821	0.5	4.9	4.31*^a^*	3.33-6.13
Pancreatic NEN	Overall	397	1.2	2.7	2.83*^a^*	1.72-4.26
	Female	164	0.5	0.5	2.59*^a^*	1.65-4.00
	Male	233	0.7	2.2	4.25*^a^*	2.79-5.60
Pulmonary carcinoid	Overall	486	0.6	2.3	2.31*^a^*	1.71-3.22
	Female	284	0.3	1.6	2.82*^a^*	1.98-4.20
	Male	202	0.4	0.7	1.32*^a^*	0.45-2.65
PPGL	Overall	51	0	0.4	2.61*^a^*	0.17-5.31
ACC	Overall	76	0.2	0.2	0.06	−1.96-2.27
MTC	Overall	64	0.3	0.5	0.85	−0.27-2.49
GI NEN by site						
Esophageal NEN	Overall	15	0	0.2	0.19	−1.12-1.55
Gastric NEN	Overall	144	0.2	0.9	1.50	−0.24-3.25
Small intestinal NEN	Overall	626	0.9	3.0	2.14*^a^*	1.55-2.99
Colon NEN	Overall	147	0	0.7	2.21*^a^*	0.82-4.69
Rectal NEN	Overall	235	0	2.5	5.28*^a^*	3.33-8.11
Appendiceal NEN	Overall	414	0.3	4.0	5.87*^a^*	4.78-7.41

Abbreviations: AAPC, average annual percent change; ACC, adrenocortical carcinoma; ASIR, age-standardized incidence rates; CI, confidence interval; GI, gastrointestinal; MTC, medullary thyroid carcinoma; NEN, neuroendocrine neoplasm; PPGL, pheochromocytoma/paraganglioma.

^a^Indicates statistically significant.

Then, a subgroup analysis of gastrointestinal NENs based on the anatomical site of the tumor was performed (Table S2 [16]). From 1980 to 2022, no significant changes in AAPC in esophageal and gastric NENs were observed with an ASIR of 0.2 and 0.9 per 100,000 in 2022, respectively. However, a significant increase in ASIR from 1980 to 2022 was observed for small intestinal NEN (from 0.9 to 3 per 100,000, AAPC 2.14%, 95% CI 1.55-2.99%), colon NEN (from 0 to 0.7 per 100,000, AAPC 2.21%, 95% CI 0.82-4.69%), rectal NEN (from 0 to 2.5 per 100,000, AAPC 5.28%, 95% CI 3.33-8.11%), and appendiceal NEN (from 0.3 to 4 per 100,000, AAPC 5.87%, 95% CI 4.78-7.41%) (Table 2). For rectal NENs, the increase in ASIR was most pronounced after 2006, whereas the increase in ASIR was strongest for appendiceal NEN after 2009, representing the highest ASIR of all gastrointestinal NENs with a maximum incidence rate of 4.4 per 100,000 in 2019 (Fig. 1B).

In pancreatic NENs, an increasing trend in ASIR was observed during the study period. However, Jointpoint regression analysis revealed a significant rise in pancreatic NENs mainly from 2004 to 2022, from 0.6 to 2.7 per 100,000 (APC 5.36%, 95% CI 3.13-16.97%) (Fig. 1C). The rise in pancreatic NENs was more pronounced for males (AAPC 4.25%, 95% CI 2.79-5.60%) than for females (AAPC 2.59%; 95% CI 1.65-4.0%).

### Other NENs and Other Endocrine Tumors

In pulmonary carcinoid, a significant increase of the incidence rate from 0.6 in 1980 to 2.3 per 100,000 in 2022 (AAPC 2.31%; 95% CI 1.71-3.22%) was observed, with a more pronounced AAPC in females (2.82%; 95% CI: 1.98-4.20%) than in males (1.32%; 95% CI: 0.45-2.65%) (Fig. 1D).

In PPGLs, ASIR significantly increased with an AAPC of 2.61% (95% CI 0.17-5.31%) from 0 in 1980 to 0.4 per 100,000 in 2022. The increase was most pronounced after 2015. In contrast, ASIR of MTC (AAPC 0.85%, 95% CI −0.27-2.49%) and ACC (AAPC 0.06%, 95% CI −1.96-2.27%) did not increase significantly between 1980 and 2022 (Fig. 1E).

### ASIR based on Histologic Subtypes

Over the observation period, the ASIR of NET G1 increased markedly from 1.8 per 100,000 in 1980 to 11.4 per 100,000 in 2022, representing the primary contributor to the overall rise in incidence. The ASIR of NECs also increased, although more modestly, and started to decline after 2010. Sites of NECs are presented in Table S3 [16]. In contrast, the incidence of MiNENs remained stable across the entire observation period. Notably, the ASIR of other NENs, including functional pancreatic NETs, decreased over the study period (Table 3, Fig. 1F).

**Table 3.** Summary of ASIR and AAPC for selected histologic subtypes of NENs (1980-2022)

Tumor type	Group	ASIR 1980 (per 100,000)	ASIR 2022 (per 100,000)	AAPC (%)	95% CI (%)
NET G1	Overall	1.8	11.4	3.87*^a^*	3.16-4.87
NET G2/3	Overall	0.1	2.5	20.43*^a^*	14.17-43.84
NEC	Overall	0.5	2.2	5.61*^a^*	3.96-9.36
Other NEN	Overall	0.9	0.1	−5.54	−10.5–−3.66
MiNEN	Overall	0.1	0.2	−1.08	−5.68-2.94

Abbreviations: AAPC, average annual percent change; ASIR, age-standardized incidence rates; CI, confidence interval; MiNEN, mixed neuroendocrine–nonneuroendocrine neoplasms; NEC, neuroendocrine carcinoma; NEN, neuroendocrine neoplasm; NET, neuroendocrine tumors.

^a^Indicates statistically significant.

### Survival Analyses

**Figure 2.**
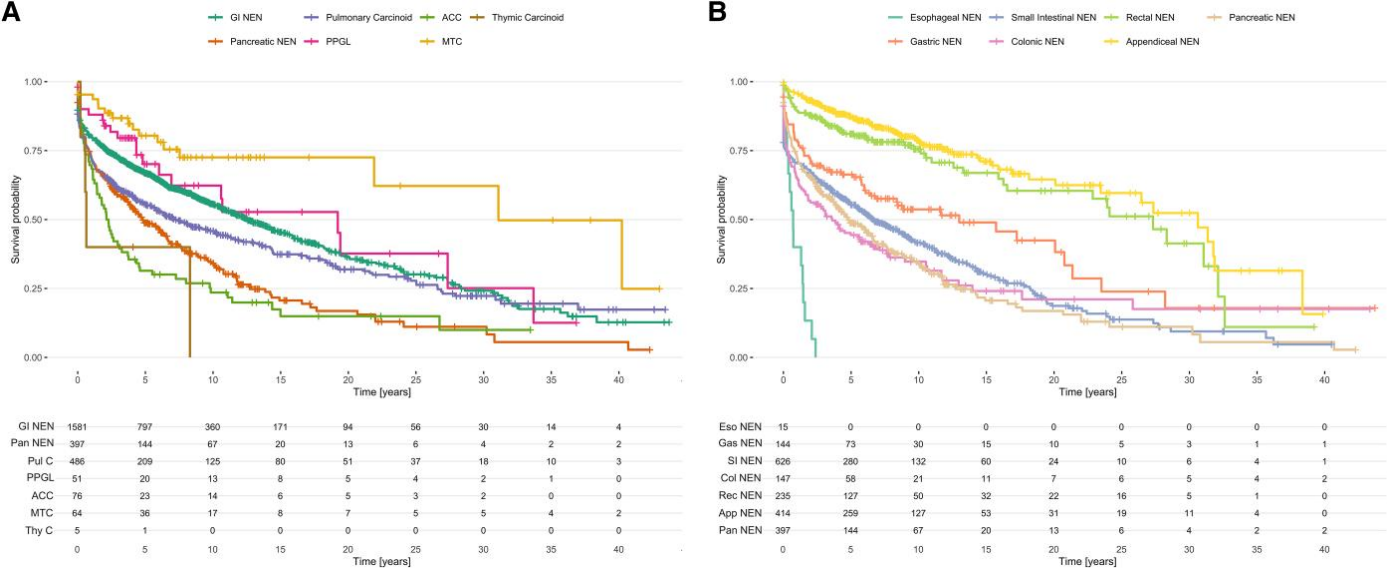
Kaplan–Meier survival curves stratified by NENs sites and ACC. Overall survival in patients with NENs stratified by broader tumor category (A) and gastrointestinal NENs stratified by anatomical site (B). Tables below the figure show number at risk. Abbreviations: ACC, adrenocortical carcinoma; MTC, medullary thyroid carcinoma; NEN, neuroendocrine neoplasm; PPGL, pheochromocytoma/paraganglioma.

Kaplan–Meier survival analysis was performed for the subgroups of NENs based on tumor entity and site. Evidence of a statistically significant difference in median OS between tumor subtypes was found (log-rank test, *P* < .001). Median OS was 12.6 years (95% CI 11.2-14.3) for gastrointestinal NENs, 4.8 years (95% CI 3.9-6.4) for pancreatic NENs, 7.1 years (95% CI 5.6-10.3) for pulmonary carcinoids, 19.2 years [95% CI 6.9-not reached (NR)] for PPGLs, 2.2 years (95% CI 1.43-3.7) for ACCs, 31.1 years (95% CI 21.9-NR) for MTCs, and 0.6 years (95% CI 0.6-NR) for thymic carcinoids (Fig. 2A, Table S4 [16]).

Next, the median OS of gastrointestinal NENs stratified by site was analyzed. Again, median OS differed significantly between NEN subtypes with esophageal NENs having the shortest OS (median 0.7 years; 95% CI 0.4-1.6) followed by NENs of the colon (median OS 3.6 years; 95% CI 1.8-6.6). Patients with NENs of the small intestine had a median OS of 6.7 years (95% CI 5.6-8.6) and those with gastric NENs of 13 years (95% CI 6.8-21.4). The longest median OS was observed in patients with rectal (median OS 27.3 years; 95% CI 2.9-NR) and appendiceal NENs (median OS 30.4 years; 95% CI 26.5-NR) (Fig. 2B).

The median OS in patients with NET G1 was significantly longer with 17.9 years (95% CI 14.6-21.8) compared to the median OS of patients with NET G2/G3 (median OS 10.8 years; 95% CI 10.1-NR) and patients with NECs (median OS 2.7 years; 95% CI 2-3.4) (Fig. S1 [16]).

## Discussion

Here we report the ASIRs and survival of patients with NENs stratified by subgroups over 4 decades based on data from the cancer registry of the Canton of Zurich, representing approximately 18% of the total population of Switzerland [14].

Our data shows a significant increase in ASIR over the study period for gastrointestinal, pancreatic, and pulmonary NENs, with the most pronounced increase in ASIR after 2005. For gastrointestinal NENs, the ASIR increased almost 4-fold since 2005. This increase was mainly driven by rising ASIR of rectal and appendiceal NENs after 2006. The incidence of appendiceal NENs increased from 0.3 in 1980 to 4.0 per 100,000 in 2022, corresponding to an astonishing 13.3-fold increase, whereas the incidence of rectal NENs increased 2.5-fold over the study period. Small intestinal NENs represented the most frequent site of gastrointestinal NENs in our cohort. Within this group, the rise in incidence has been steady since 1980, with an AAPC of 2.14% and a 3-fold increase in incidence from 1980 to 2022. A rise in the incidence of pancreatic NENs was again most pronounced after 2004, with an APC of 5.36% mainly driven by an increase in ASIR in male patients.

Globally, an increase in NEN incidence has been reported since the early 2000s [6-10]. Data from the Bavarian cancer registry in Germany showed a doubling of GEP-NEN incidence from 2.2 to 4.8 per 100,000 between 2005 and 2019 with the most pronounced increase for NENs of the stomach, followed by those of the appendix, pancreas, and rectum [7]. In contrast, we only observed a statistically significant increase in ASIR in gastric NENs between 2009 and 2013 but not over the total study period. A study from western Switzerland, using data from cancer registries of the Cantons of Vaud and Neuchâtel (1976–2016), reported a lower average annual increase in GEP-NEN incidence (1.7% in men and 1.3% in women) compared to our data from the more populous Zurich region [12].

In the Netherlands and Norway, NEN incidence more than doubled over 2 decades, reaching 4.9 (2010) and 9.97 (2021) per 100,000, respectively [8, 21]. In contrast, data from Iceland indicate a stable and, in comparison to our data, lower incidence of both small intestinal NENs (0.78 per 100,000) and GEP-NENs (3.65 per 100,000) over a 30-year period [22, 23].

According to the data from the National Cancer Institute's Surveillance, Epidemiology, and End Results (SEER)program in the United States, GEP- and pulmonary NEN incidence increased 5.2-fold between 1975 and 2021, reaching up to 8.52 per 100,000 (2021) with the prominent increases observed in appendiceal NENs, well-differentiated NENs, and a shift toward earlier-stage diagnoses [6, 11, 13]. In line with this, we also observed the strongest increase in the incidence of appendiceal NENs. In contrast to the SEER database, we observed a higher ASIR for small intestinal NENs compared to pulmonary NENs at the end of the study period in the canton of Zurich. In Australia, NEN incidence tripled to 6.3 per 100,000 between 1986 and 2015 with stable mortality, while in Japan, the first national registry-based analysis reported an age-adjusted overall incidence of 3.53 per 100,000 in 2016, which predominantly occurred in the rectum and pancreas [9, 24]. Hence, in line with our results, epidemiological studies in Europe, America, and Asia found an increase in diagnosis of NENs of most organ systems [6-8, 10].

It remains unclear, however, whether the observed rise in NEN incidence reflects improved detection methods, including more incidental findings, shifts in risk factors such as a potential correlation with the rise of prevalence of metabolic diseases, or an increase in exposure to yet unknown risk factors [25-29]. Based on our data, the most pronounced increase in ASIR occurred after 2005 and was primarily driven by a rise in NET G1, a subtype often asymptomatic and frequently detected incidentally on computed tomography or magnetic resonance imaging performed for unrelated indications or during screening colonoscopy in the case of rectal and colonic NENs [30]. Moreover, functional imaging became available, and endoscopic ultrasound techniques have improved substantially over the past decade [31, 32]. The hypothesis of early, incidental detection is further supported by the observation that symptomatic NENs, such as those of the esophagus, stomach, and functional pancreatic NETs, did not increase at a similar rate over the last decade. Similarly, ACC, in which patients typically present due to cortisol or androgen excess, also showed no significant increase in incidence in our study.

However, technical advancements do not fully explain the striking increase in appendiceal NENs, a trend also observed in Bavaria [7], as these tumors are typically not detected through colonoscopy. Recent data from Switzerland indicated that appendectomies increased from 2013 to 2023 [33]. In the current study, the increase in appendiceal NENs was observed in both males and females, suggesting that the rise cannot be solely attributed to incidental findings during laparoscopic procedures, such as those performed for appendicitis or endometriosis. Prior to the introduction of ICD-O-3 at the cancer registry of the Canton of Zurich in 2003, certain NENs such as appendiceal NENs were sometimes classified as borderline or nonmalignant and were therefore not systematically captured in the cancer registry. With the implementation of ICD-O-3, these entities began to be uniformly coded as malignant, leading to more consistent inclusion of cases in the registry. Consequently, at least part of the observed increase in incidence may reflect improved case ascertainment and changes in coding definitions rather than a true rise in disease occurrence. In line with this, the reclassification of appendiceal NENs as malignant has, according to a study from the United States, led to an artificial increase in reported colorectal cancers in children and young adults [34].

The more gradual increase in pancreatic and small intestinal NENs is, however, not fully explained by the factors discussed here. A potential association with the rising prevalence of metabolic disorders appears plausible, particularly given epidemiological evidence linking obesity and diabetes to increased risk of several malignancies, including colorectal, breast, endometrial, and liver cancers and NENs [25-28, 35]. If insulin signaling plays a role in tumorigenesis, it is important to consider that local insulin concentrations are higher in the pancreas and liver than in the systemic circulation. NENs arising in these regions, including those with liver metastases, may thus be especially vulnerable to the effects of hyperinsulinemia due to their anatomical proximity to insulin-secreting β cells. Several authors have proposed that type 2 diabetes and obesity may increase the risk of pancreatic and small intestinal NENs [26, 27, 36, 37]. In addition, the presence of metabolic syndrome, nonalcoholic fatty liver disease, and visceral adiposity has been associated with more adverse clinicopathological features in patients with GEP-NENs [28].

Notably, our data also show an increase in the ASIR of PPGLs after 2015 from 0.2 to 0.4 per 100,000 in 2022. This rate remains lower than reported in studies from Denmark and Canada, which estimated an incidence of 0.66 cases per 100,000 person-years in the general population [38, 39]. The incidence of MTC (ASIR 2022 0.5 per 100,000) and ACC (ASIR 2022 0.2 per 100,000) did not change significantly over the last 4 decades in Zurich. ACC incidence was though higher compared to data from the Dutch Cancer and Danish health registries, both reporting an incidence rate from 0.1 to 0.14 per 100,000 [40, 41].

We also calculated the median OS of NEN patients. The shortest median OS was seen for thymic carcinoid (0.6 years) and ACC (2.2 years), as expected. Of note, the gastrointestinal NENs with the most pronounced increase in ASIR were also those with the longest median OS. Rectal NENs had a median OS of 27.3 years and appendiceal NENs a median OS of 30.4 years. This is in line with recent data from the SEER program, where patients with rectal and appendiceal NENs had the longest survival of over 30 years [11]. This finding supports the hypothesis that rising incidence reflects increased early detection, with tumors often identified at localized stages and effectively treated by endoscopic or surgical resection, resulting in long-term remission.

A limitation of this study is the limited number of cases in certain subgroups. Small subgroup sizes and incomplete information on stage and treatment also precluded age-specific and subtype-specific analyses. An analysis of canton-specific cancer registries only covers a part of Switzerland's population, while a nationwide program would provide more comprehensive information. While we acknowledge the statistical constraints associated with such analyses, particularly the potential for fluctuations in incidence trends due to single-case fluctuations, we choose to maintain separate reporting for rare entities like PPGL and ACC.

In conclusion, our study adds more population-based data on NEN incidence up to 2022 by incorporating more recent data over a very long observation period (>40 years) [6, 42]. We provide a comprehensive analysis of both common and rare NENs, including rare endocrine tumors such as PPGLs, MTC, and the non-NEN entity ACC, which are often underrepresented in population-based studies. We confirm and extend the previously observed trends of continuously rising incidence of NENs through 2022. This increase was evident across nearly all anatomical sites, with the most pronounced increase observed in rectal and appendiceal NENs, while the incidence of gastric and esophageal NENs remained stable or declined. Notably, the incidence of GEP-NENs and PPGLs continues to rise, whereas that of MTC and ACC remained stable over the 43-year observation period. These findings reinforce the ongoing shift in the epidemiology of NENs and underscore the importance of continued research into the underlying risk factors contributing to their development.

## Data Availability

Restrictions apply to the availability of some or all data generated or analyzed during this study to preserve patient confidentiality or because they were used under license. The corresponding author will on request detail the restrictions and any conditions under which access to some data may be provided.
